# Predictive control of musculotendon loads across fast and slow-twitch muscles in a simulated system with parallel actuation

**DOI:** 10.1017/wtc.2025.1

**Published:** 2025-02-20

**Authors:** Mahdi Nabipour, Gregory S. Sawicki, Massimo Sartori

**Affiliations:** 1 Neuromuscular Robotics Laboratory, Department of Biomechanical Engineering, University of Twente, Enschede, the Netherlands; 2 Human Physiology of Wearable Robotics (PoWeR) laboratory, George W. Woodruff School of Mechanical Engineering, School of Biological Sciences and Institute for Robotics and Intelligent Machines, Georgia Institute of Technology, Atlanta, GA, USA

**Keywords:** locomotion, walking, hopping, muscle fiber phenotype, ankle plantarflexors, achilles tendon, musculotendon unit, hill-type muscle, predictive force control, injury prevention, wearable robotics

## Abstract

Research in lower limb wearable robotic control has largely focused on reducing the metabolic cost of walking or compensating for a portion of the biological joint torque, for example, by applying support proportional to estimated biological joint torques. However, due to different musculotendon unit (MTU) contractile speed properties, less attention has been given to the development of wearable robotic controllers that can steer MTU dynamics directly. Therefore, closed-loop control of MTU dynamics needs to be robust across fiber phenotypes, that is ranging from slow type I to fast type IIx in humans. The ability to perform closed-loop control the in-vivo dynamics of MTUs could lead to a new class of wearable robots that can provide precise support to targeted MTUs for preventing onset of injury or providing precision rehabilitation to selected damaged tissues. In this paper, we introduce a novel closed-loop control framework that utilizes nonlinear model predictive control to keep the peak Achilles tendon force within predetermined boundaries during diverse range of cyclic force production simulations in the human ankle plantarflexors. This control framework employs a computationally efficient model comprising a modified Hill-type MTU contraction dynamics component and a model of the ankle joint with parallel actuation. Results indicate that the closed-form muscle-actuation model’s computational time is in the order of microseconds and is robust to different muscle contraction velocity properties. Furthermore, the controller achieves tendon force control within a time frame below 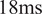 , aligning with the physiological electromechanical delay of the MTU and facilitating its potential for future real-world applications.

## 1. Introduction

Recent efforts in lower limb wearable exoskeleton and exosuit control have largely focused on two main objectives: reducing metabolic energy expenditure during locomotion (Zhang et al., 2017; Witte et al., 2020; Slade et al., 2022) and compensating for a portion of biological joint torque via, for example, neuromusculoskeletal models, or regression techniques (Durandau et al., 2022). However, in recent literature, there has been a growing focus on controlling Achilles tendon force through open-loop methods (Schmitz et al., 2022). Another approach involves leveraging Soleus contraction dynamics to provide assistive force, aiming to lower metabolic expenditure (Nuckols et al., 2021). Notably, the required processing time for this study was considerably shorter than the fastest human-in-the-loop (HIL) optimization used for minimising metabolic expenditure of walking (Slade et al., 2022). However, the skeletal musculotendon unit (MTU) undergoes remarkable adaptations in response to mechanical stimuli (Wisdom et al., 2015), which is neglected in the considerations of current controllers.

Human skeletal muscle consists of three primary types of muscle fibers classified into slow (type I) and fast (types II-a, and II-x) according to their specific myosin heavy chain isoforms (Staron, 1997). Slow-twitch fibers are fatigue-resistant, producing force slowly but steadily, ideal for endurance activities; fast-twitch fibers generate force rapidly but fatigue quickly, suited for explosive movements. Slow-twitch fibers have slower contraction velocity and are recruited first during low-intensity tasks, while fast-twitch fibers contract more rapidly and are activated for high-force demands. These differences in force–velocity (F–V) relation, response to activation changes, and contractile properties underscore the distinct roles and adaptations of muscle fiber types in various physiological and performance contexts (Bottinelli and Reggiani, 2000). In addition, muscle fibers have the ability to adapt to varying demands by altering their size or composition. This adaptability forms the physiological foundation for many physical therapy techniques aimed at enhancing a patient’s muscle force generation or endurance (Scott et al., 2001). Moreover, variations in fiber type composition may contribute to some of the impairments and disabilities observed in patients Pette (Pette and Staron, 1997).

Existing control schemes provide assistive torques to biological joints with no knowledge on the effect on joint’s constituent MTU force. The development of wearable robots capable of achieving direct closed-loop control over the mechanical loads applied to individual MTUs, while being robust to changes in muscle fiber type, has not yet been fully addressed. Such advancements would enable wearable robots to offer bio-protective support, reducing the risk of musculoskeletal injuries or tissue maladaptation associated with repetitive intense loads. This protection would be particularly beneficial during transitions in muscle fiber composition caused by injury, recovery, aging, or disease, helping to prevent tissue catabolic function in these critical phases. Similarly, this new class of wearable robots could deliver precise mechanical stress and strain to injured tissues to facilitate stress- and strain-induced tissue regeneration following injury, opening to precision robot-aided rehabilitation.

Here, we present an initial step toward addressing this gap by creating a predictive model-based control framework for closed-loop control of peak MTU load during cyclic motions such as hopping. To accomplish this goal, we implement nonlinear model predictive control (NMPC) in the control architecture due to its intrinsic robustness and predictive capabilities. NMPC predicts future states of the system, proactively preventing the MTU load from surpassing the threshold. Our objective is to regulate the peak Achilles tendon force by integrating a simulated exoskeleton in parallel with the ankle plantarflexors. Furthermore, we will assess the robustness of this controller to variations in muscle fiber phenotypes.

For the model-based predictive control framework, we need a combined MTU-exoskeleton model in ordinary differential equation (ODE) form. We opt for a conventional Hill-type muscle model (Caillet et al., 2022) with parallel damping primarily due to their efficiency and simplicity in parameterization, as opposed to alternatives such as Huxley’s models of muscle contraction (Huxley and Hanson, 1954; Huxley and Niedergerke, 1954). In a prior preliminary study, we employed univariate linear regression to develop a closed-form solution for the damped Hill-type muscle model (Nabipour et al., 2023; Nabipour and Sartori, 2023). Nevertheless, the resulting model did not exhibit generalizability across various locomotion speeds and different muscles. Consequently, we propose a computationally efficient simplified closed-form solution for the damped Hill-type model to be integrated into the predictive control framework. In the existing literature, implicit modeling (De Groote et al., 2016) for the conventional model and numerical solutions (Millard et al., 2013) for the damped Hill-type models are available.

In this paper, we begin by introducing a straightforward closed-form ODE model for the combined human lower limb and exoskeleton system. Next, we modify the force-velocity equation to account for variations in muscle fiber phenotypes. We then apply the predictive control framework to keep the Achilles tendon force within limits. Our analysis also explores different control horizons for the NMPC and varying levels of support within the control framework. In addition, we assess the controller’s robustness in controlling peak tendon force across muscles with distinct twitch characteristics.

## 2. Methods

Below, we explain in detail the process of deriving the closed-form set of ODEs for the combined MTU-exoskeleton system, focusing on hopping motion as a representative cyclic locomotion-like movement. Subsequently, an analysis of the control structure and the methodology employed for assessing the robustness and sensitivity of the control framework will be presented.

### 2.1. Modeling combined MTU-exoskeleton system

**Figure 1.**
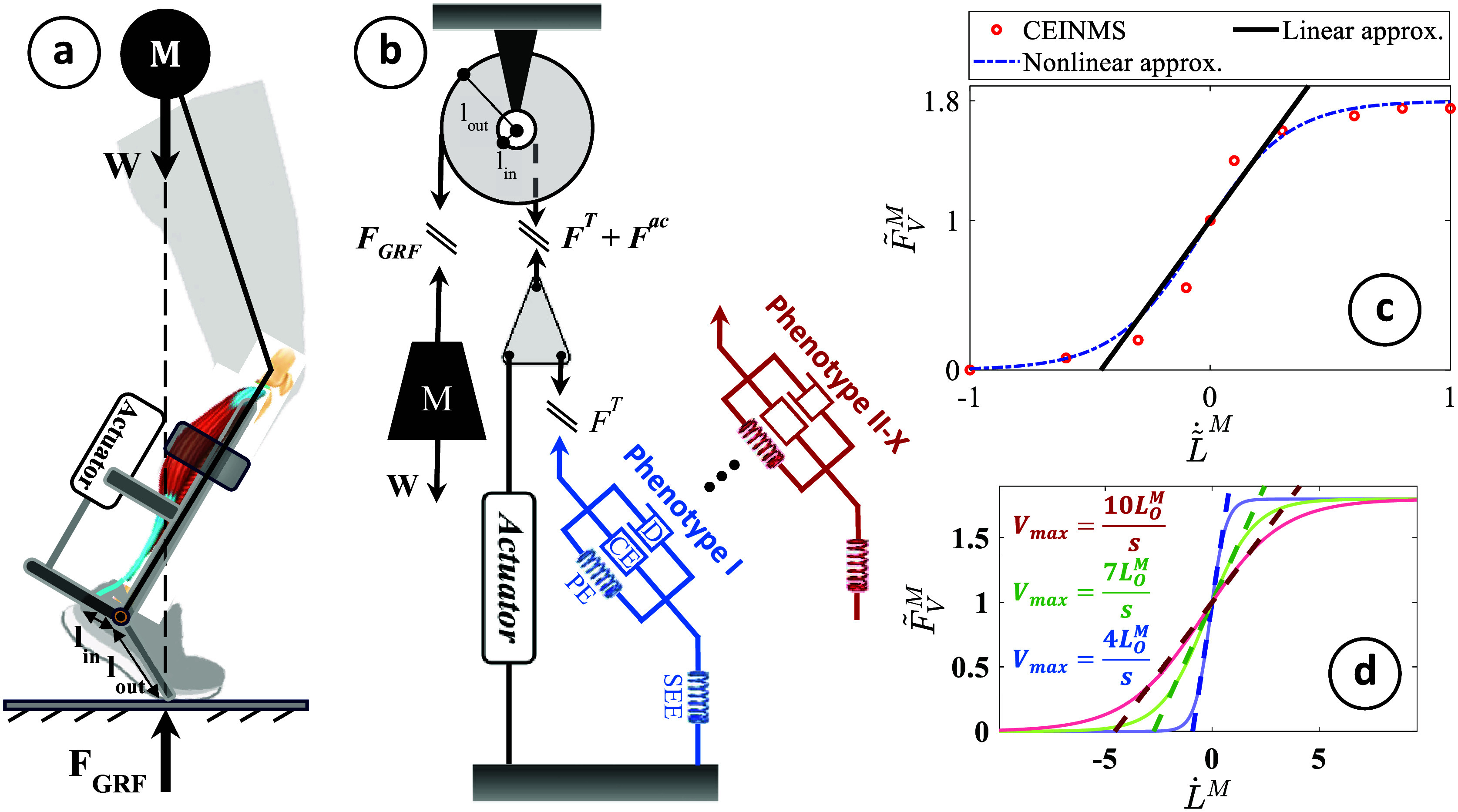
(a) The human-exoskeleton system illustrating the combined human-exoskeleton model during hopping, (b) the simplified pulley-mass model utilized for modeling a parallel exoskeleton integrated with the triceps surae during hopping, (c) the F–V relation of the MTU contractile element acquired through CEINMS, incorporating the nonlinear equation from Anderson (2007), and the linear approximation in equation 3, (d) alterations in the F–V relation transitioning from slow (in blue) to fast (in red) twitch muscle fibers.

The simulation of hopping motion involves simplifying the combined MTU-exoskeleton system into two main components: a lumped plantarflexor muscle (representing the whole triceps surae) and a parallel exoskeleton actuator capable of providing assistance parallel to the plantarflexors (Figure 1). This simplification assumes the presence of a single lumped MTU instead of individual plantarflexing MTUs (Robertson et al., 2014; Robertson and Sawicki, 2014).

**Figure 2.**
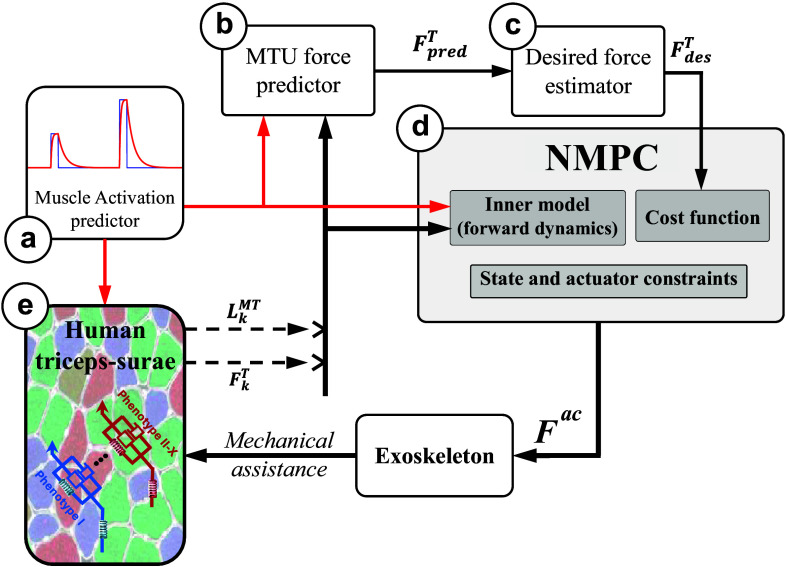
The NMPC control framework: The closed-form dynamics of the combined MTU and motion-related ODE [equations (2) and (5)] leverage the predetermined activation (a) to compute the MTU force in the tendon force predictor (b) over a horizon. In addition, the current muscle activation is fed into the human lumped triceps surae muscle model to determine the present MTU length and tendon force (e). The estimated desired tendon force (c) is derived from the predicted future MTU force (b), which is then relayed to the NMPC (d) alongside the activation over the horizon and current MTU length and force obtained from the human (e). The controller’s output is subsequently applied to the human through the exoskeleton. Consequently, due to this mechanical assistance, the muscle fiber phenotype could undergo changes (e) unbeknownst to the controller.

#### 2.1.1. Modeling lumped triceps surae

We utilize a Hill-type model incorporating a pennation angle to simulate MTU dynamics (Thelen, 2003; Caillet et al., 2022). This model conventionally comprises the contractile element of the muscle arranged in both series and parallel configurations with elastic elements. We opt for a modified version of this model, where an additional damping element is added parallel to the muscle’s contractile and parallel elastic element (see Figure 1). As a result, the tendon force is derived as:
(1)

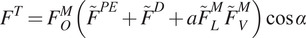

where 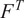 , 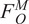 and 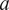 , and 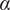 represent the tendon force, maximum isometric force, muscle activation, and pennation angle, respectively. Also, 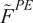 , 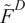 , 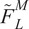 , and 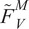 denote the normalized force of the parallel passive element, parallel damping, and contractile element’s active force–length (F–L) and force–velocity (F–V) relation. 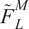 and 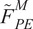 are derived from Anderson (2007). The equation governing damping is expressed as 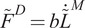 , where the coefficient 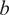 represents the damping coefficient, and 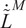 denotes the normalized muscle fiber velocity. To enable future predictions with a closed-form equation, it is often advantageous to model the system ODEs: 
(2)

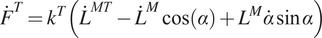

where 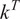 and 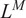 are the tendon stiffness and muscle fiber length, respectively. The stiffness can either be derived from De Groote et al. (2016) or by ploynomial fit to stiffness or tendon force-strain data points used in our previously developed Calibrated EMG-Informed Neuromusculoskeletal Modelling (CEINMS) toolbox (Pizzolato et al., 2015). 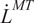 in equation (2)) is directly derived from the kinematics of the movement and represents the rate of change of MTU length. The 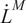 is often obtained via regression in the cases where damping is not incorporated in the Hill-type model. When damping is considered, no closed-form solutions were derived and researchers often use numerical solutions (Millard et al., 2013). The normalized muscle fiber velocity does not cross the range of 
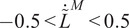
 during walking and running with various speeds, as noted by Arnold et al. (2013). Therefore, we simplify the F–V relation assuming muscle fiber contraction velocity remains within the linear range, as depicted in Figure 1:
(3)

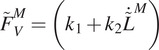



The optimal values for 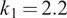 and 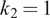 have been identified to fit the F–V curve accurately within the CEINMS toolbox. By combining equations (1) and (2), we can obtain:
(4)

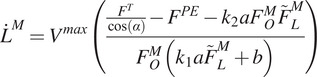

where 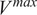 represents the maximum fiber velocity which ranges from 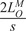 to, 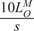 depending on whether the muscle’s constituent fiber phenotype is slow or fast-twitch (Zajac, 1989). By replacing 4 in 2, the MTU contraction dynamics in ODE form will be obtained.

Gaining direct control over individual MTU dynamics is, however, complex. MTUs display a large repertoire of dynamic behaviors, which is in part determined by the constituent muscle fiber phenotype (Wisdom et al., 2015). Skeletal muscle fibers’ sarcomeres can express a range of myosin heavy chain isoforms, from slow (i.e., type I) to fast (i.e., type IIa and IIx). This impacts the muscle’s ability to generate force per unit time. Fast-twitch muscle fibers are characterized by their ability to produce a high amount of force in a short period but are also more prone to fatigue. In contrast, slow-twitch fibers produce less force over a longer period but are highly resistant to fatigue. These characteristics, aside from fatigue, are reflected in the muscle’s F–V relationship (Figure 1d).

#### 2.1.2. Simplified model for hopping

The MTU contraction dynamics model should be combined with a motion-related equation to obtain the combined MTU-exoskeleton system. We use the same model used by Robertson et al. (2014) and Robertson and Sawicki (2014) and add a parallel damping to the MTU contractile element. In addition, we incorporate a parallel actuator force into the system to simulate the exoskeleton.
(5)

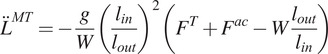



In this equation, the ratio 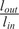 represents the mechanical advantage of the pulley-mass system shown in Figure 1. The combined MTU-exoskeleton model [equations (2) and (5)] are used as the main model (the control plant) within the control framework.

### 2.2. Tendon force control using NMPC

To effectively maintain musculotendon or joint loads below a predefined threshold while ensuring user comfort, predictive controllers are essential. These controllers can predict future trends in MTU loads and activate actuators preemptively to prevent the load from exceeding the threshold. To address this need, we integrate an NMPC algorithm within our framework. Unlike other model-based controllers, MPC algorithms offer the advantage of setting explicit limits on both the system states and controller outputs. The primary objective in our optimization problem is to minimize the following cost function while ensuring that the tendon force remains within the specified limits.
(6)

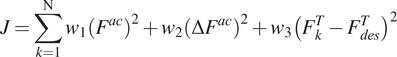



Here, 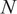 represents the control horizon, while the weights 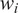 determine the influence of tendon force, actuator force ( 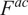 ), and its increment ( 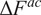 ) on the overall cost function. Specifically, 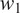 penalizes high actuator force, thereby reducing power consumption. 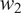 penalizes large fluctuations in actuator force, promoting smoother, more gradual changes in actuation. Finally, 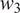 penalizes the deviation between the actual and desired Achilles tendon force during hopping. We use a decreasing horizon and the smallest permissible horizon in our simulation is considered to be equal to 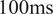 (Nabipour et al., 2023). In all our simulations, we operate under the assumption that we have foreknowledge of the future MTU activation. This assumption remains valid particularly in cyclic tasks like hopping, where muscle synergies enable us to infer the activation throughout the entire cycle.

### 2.3. Robustness of the control framework

#### 2.3.1. Different horizons and levels of support

The initial prediction control horizon determines the duration over which the control framework predicts future MTU force profiles to decide whether to activate the NMPC. In addition, as previously discussed, predicted future MTU force also serves as the starting point for the decreasing control horizon of the NMPC algorithm. Therefore, an excessively large initial control horizon can lead to significant time consumption, while overly small values may impair the controller’s ability to maintain the MTU load below a predetermined threshold. To analyze the sensitivity of the NMPC to this control parameter, we will simulate various initial horizons ranging from 100 to 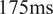 .

Having a controller robust to changes in levels of support ensures the adaptability of the exoskeleton to different environments and enhances the user’s comfort and safety. We will simulate variations in support levels by maintaining a fixed initial horizon and changing the threshold of the NMPC algorithm. On the controller side, this essentially involves altering the 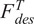 value. Adjusting the threshold prompts the controller to target matching the maximum MTU load to the specified threshold value. Consequently, modifying the threshold is equivalent to adjusting the desired force.

#### 2.3.2. Controller robustness to various muscle phenotypes

The investigation into the robustness of the control framework to changes in muscle fiber phenotypes is conducted in two ways: (a) when the controller is informed of the phenotype change and (b) when the controller is unaware of the change. In all scenarios, a constant tendon force threshold of 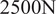 is maintained. When the controller is aware of the phenotype change the 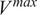 in the hopping model and the controller have the same value. It should be noted that the tendon force profile changes due to change in the phenotype of the muscle fiber.

Therefore, we simulate various fast and slow-twitch muscle phenotypes by tuning the parameter 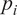 in the equation 
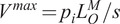
 within the MTU model. In our simulations, we adjust this parameter across values of 2 or 4 (representing slow-twitch muscle fiber), 7, and 10 (representing fast-twitch muscle fiber) (Zajac, 1989). The controller’s ability to handle various phenotypes will be examined by simulating its behavior during hopping at a frequency of 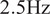 , both when the controller is aware and unaware of the change in muscle fiber phenotype. In the cases that the controller is aware of the change, the 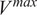 in the hopping model (Figure 2e) and the framework (the future predictor and the NMPC inner model, (Figure 2b and 2d) are the same.

## 3. Results

### 3.1. Modeling

**Figure 3.**
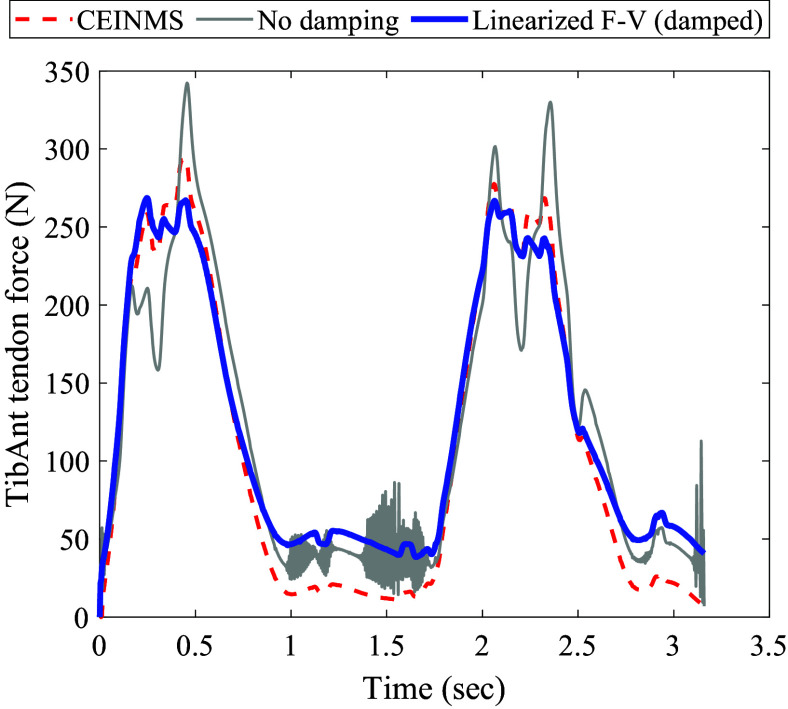
Comparison of muscle force estimation methods during plantar/dorsiflexion of the Tibialis Anterior muscle on a dynamometer: The muscle force estimated by CEINMS is represented by the red dotted line. The gray line shows the muscle force estimation using a non-damped Hill-type muscle model. The solid blue curve illustrates the muscle force estimated by the damped-linearized model.

When simulating MTU dynamics using the conventional non-damped Hill-type muscle model, the tendon force solution becomes unstable whenever muscle activation approaches zero. By introducing damping in parallel to the contractile element and linearizing the force–velocity relation to derive a closed-form analytical solution, the instability concern is effectively addressed. The muscle modeling technique was validated using data from prior studies where dorsi- and plantarflexion EMGs and kinematic data were acquired using a dynamometer. This validation process was crucial as it allowed us to confirm the accuracy of our model based on established datasets (Cop et al., 2019). Furthermore, this adjustment leads to a notable decrease in the root mean square error (RMSE) from 10.4% to 7.9% when compared with the non-damped model (see Figure 3).

### 3.2. Predictive control

#### 3.2.1. Different horizons and levels of support

**Figure 4.**
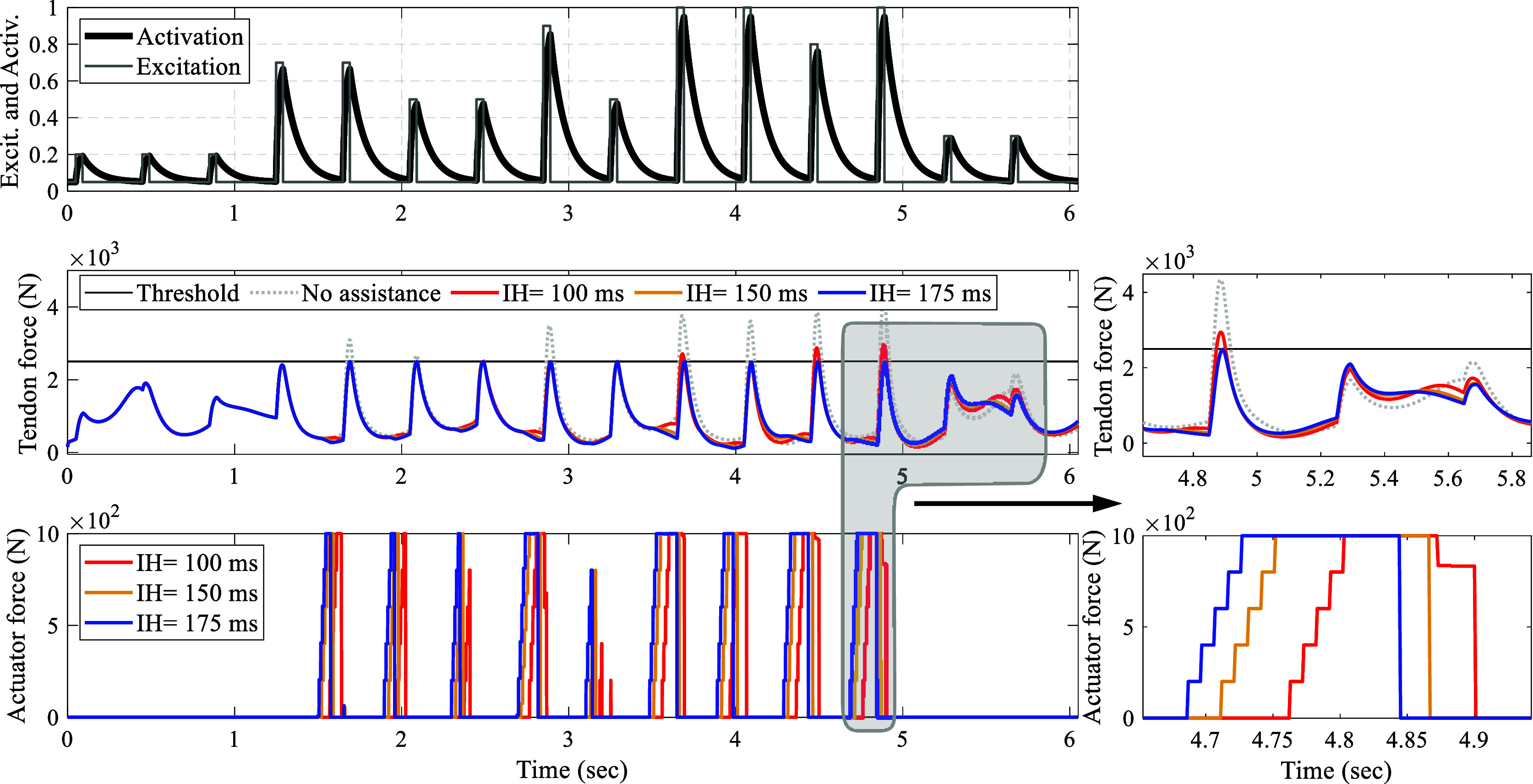
NMPC performance across varying initial control horizons: Moving from top to bottom, muscle excitation is represented by square waves with a 10% duty cycle and varying amplitudes, all maintaining a frequency of 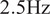 . The muscle activation dynamics are captured by a first-order differential equation. In the center, the tendon force profiles are displayed for different initial horizons (IH), alongside the scenarios without assistance and the constant predefined 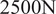 tendon force threshold. At the bottom, the actuator forces corresponding to different IHs are displayed.

Since the horizon varies in our framework, the initial horizon is of great significance. In Figure 4, the NMPC perfomance for different initial horizons starting from 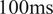 up to 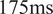 is depicted.

**Figure 5.**
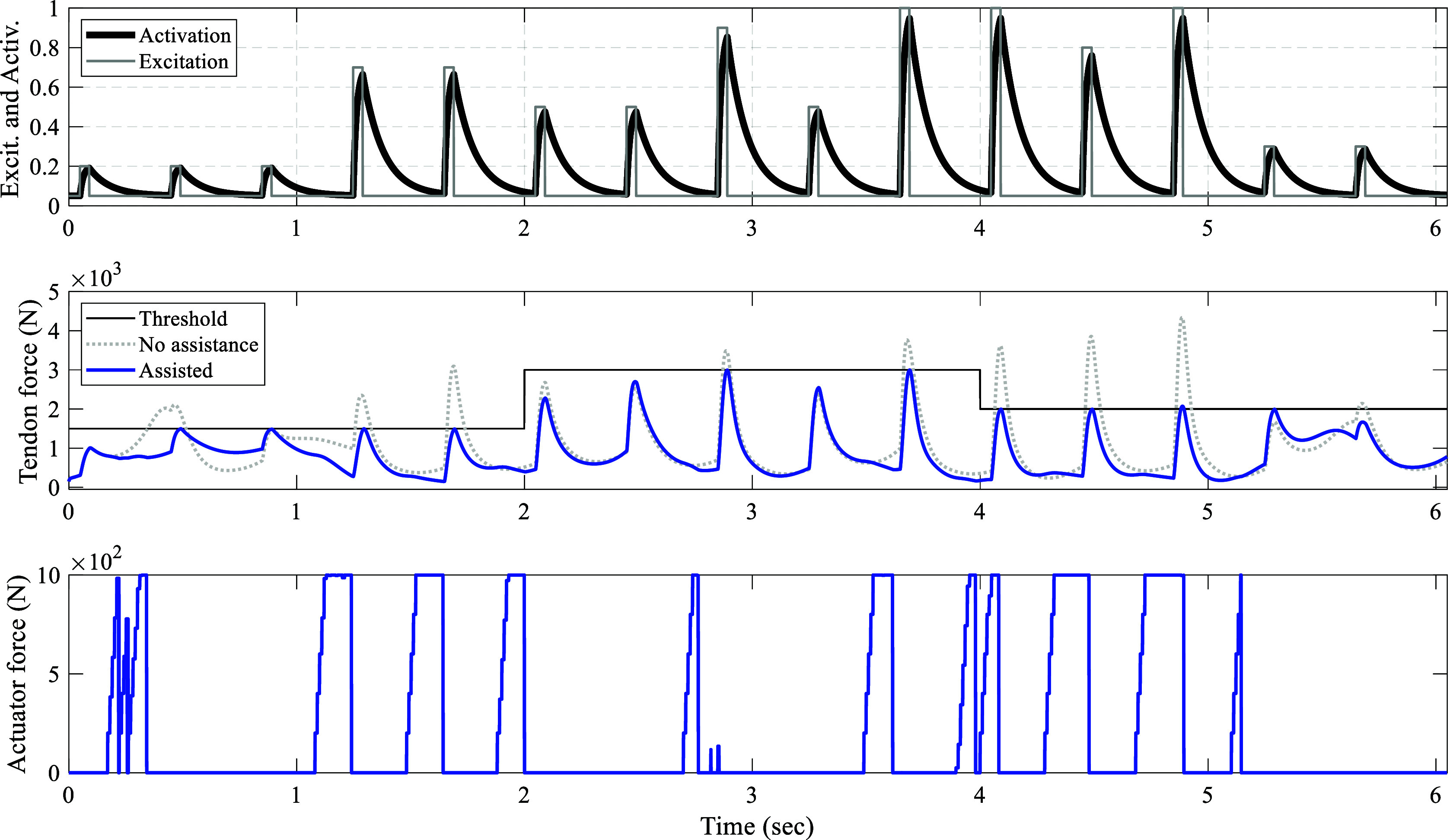
Performance of the control framework for various support levels: Progressing from top to bottom, the muscle excitation and its corresponding activation are depicted. In the middle, the Achilles tendon force is illustrated, with the unassisted scenario represented by a gray dotted line and the assisted tendon force by a solid blue line. At the bottom, the controller output required to achieve this behavior is displayed.

The hopping frequency maintained consistently throughout this study and did not change in response to assistance. This frequency is fixed at 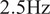 , an average value observed in the existing literature as a preferred hopping frequency (Farley et al., 1991; Funase et al., 2001; Mohammadi Nejad Rashty et al., 2024). Furthermore, the excitation and activation dynamics are based on the findings presented in Robertson et al. (2014). The maximum tendon force during simulation of hopping at a frequency of 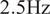 without assistance occurs when the muscle excitation amplitude is set to 1. In this scenario, the maximum tendon force reaches 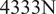 . However, when assistance is introduced with a 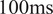 initial horizon, the maximum tendon force is reduced to 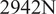 . The normalized RMSE of the maximum tendon forces achieved in every hop is calculated to be 8.2%. The median and standard deviation (STD) of the maximum tendon forces are 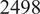 and 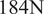 , respectively. In instances where control horizons of 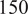 and 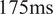 were tested, the controller consistently maintained the maximum tendon force below the threshold. However, when employing a control horizon of 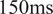 to modulate the assistance level from 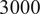 to 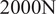 (Figure 5), it was noted that the tendon force surpassed the threshold during the transition. Consequently, in subsequent simulations, the control horizon of 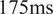 is adopted. Nonetheless, the maximum, median, and standard deviation values for a 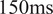 horizon are 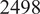 , 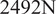 , and 4.3%, respectively. For a 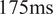 horizon, these values are 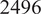 , 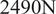 , and 4.3%, respectively.

**Figure 6.**
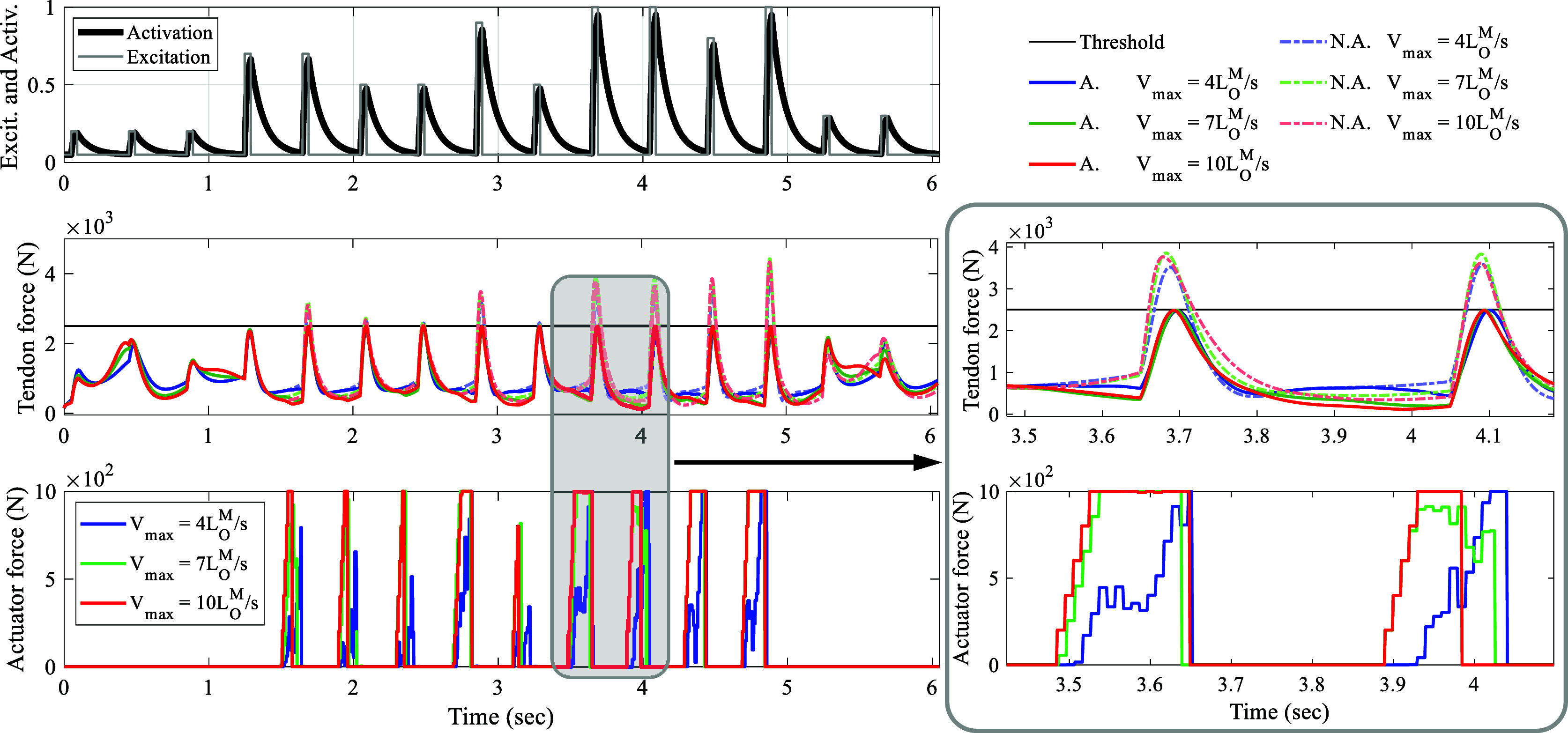
Predictive control performance applied to different muscle fiber types when the controller is aware of the fiber type. Progressing from top to bottom, the muscle excitation and its corresponding activation are illustrated. In the center, he assisted Achilles tendon forces when the controller is informed about the muscle fiber type are displayed. At the bottom, the respective control output for each muscle fiber type control is shown.

**Figure 7.**
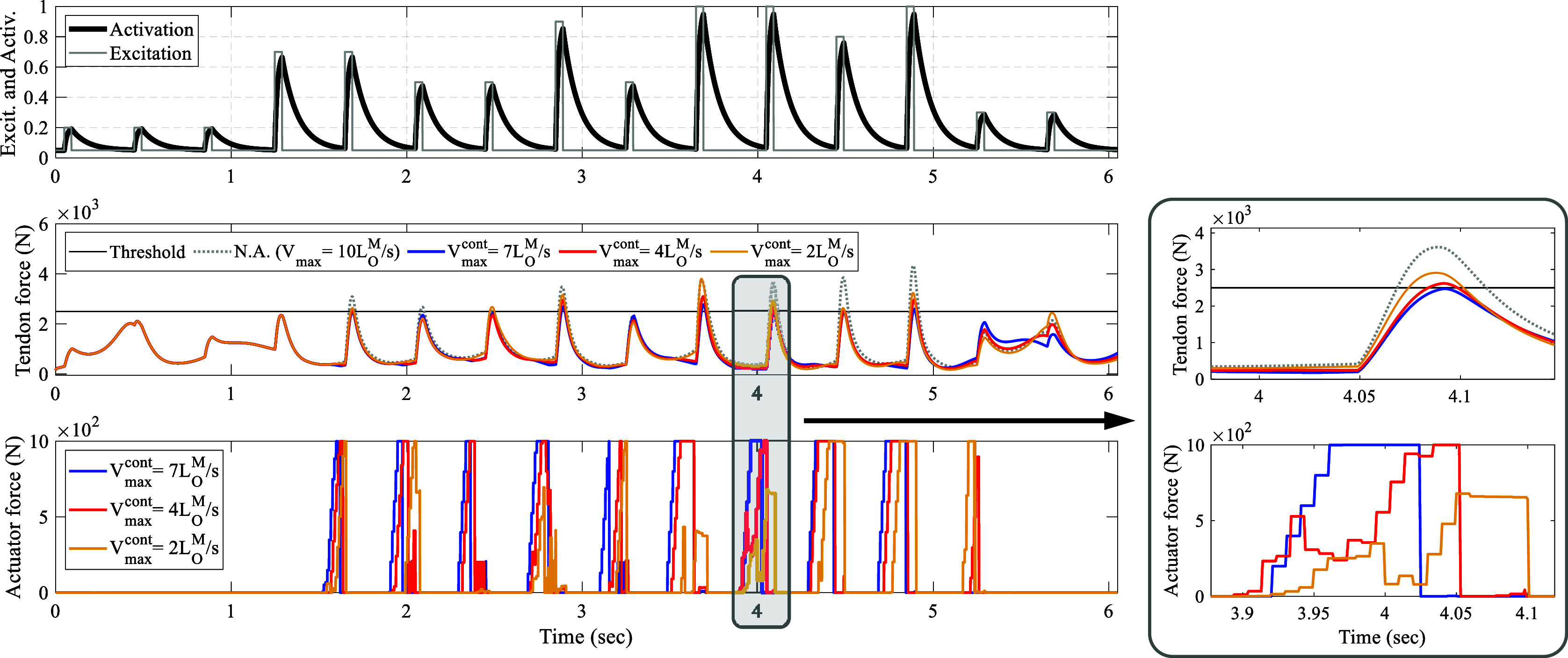
NMPC’s robustness to variations in muscle fiber type when uninformed of changes: progressing from top to bottom, the muscle excitation and its corresponding activation are illustrated. In the middle, the assisted Achilles tendon force is shown while the muscle’s fiber phenotype in the model (Figure 2e) remains constant with 
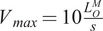
 . The controller is unaware of this value and assumes different fiber velocities. At the bottom, the controller’s output for various assumptions of muscle fiber phenotypes is displayed.

The controller’s robustness to support different levels of assistance is also investigated, as shown in Figure 5. To demonstrate various levels of support, the threshold of the NMPC algorithm is adjusted every few hop, with an initial prediction horizon of 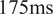 . As depicted in Figure 5, the controller dynamically adjusts the control output to ensure that the peak tendon force remains below the threshold, achieving an RMSE of 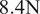 .

#### 3.2.2. Robustness to changes in muscle fiber phenotype

In this study, concerning the distinct muscle fiber phenotypes analyzed, the controller successfully keeps the peak Achilles tendon force below the threshold when it is informed about the variations in the muscle fiber phenotypes. For the type I muscle fiber ( 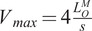 ), the average peak tendon force, STD, and normalized RMSE are 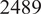 , 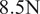 , and 0.5%, respectively. Similarly, for type II-A, these values are close at 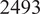 , 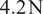 , and 0.36%, and for type II-X, they stand at 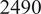 , 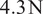 , and 0.4%, respectively. Furthermore, as observed, the non-assisted peak tendon force rises with the increase in the muscle fiber twitch velocity. Consequently, the faster twitch muscle fibers necessitate greater exoskeleton actuation to ensure that the assisted tendon force remains below the threshold.

The investigation into the robustness of the control framework was also conducted without informing the controller of the changes occurring in the muscle fiber phenotype (see Figure 7). In this scenario, the 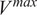 parameters in both the main model and the controller were varied with different values. It was noted that when the 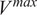 parameter in the human triceps surae model surpassed that of the controller, the controller effectively managed to produce outputs that kept the peak tendon force near the specified limit. However, a different scenario unfolded when the controller erroneously assumed a faster muscle fiber type than the actual case, resulting in assisted tendon forces falling below the threshold but significantly under the expected level, potentially compromising user comfort in practical applications.

For instance, as illustrated in Figure 7, in the case where 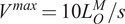 in the model and 
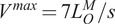
 in the controller (represented by the blue lines in Figure 7), the maximum peak controlled tendon force was 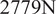 . For the case where 
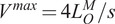
 and 
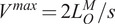
 in the controller (represented by red and yellow lines in Figure 7), these statistics take the value of 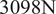 and 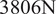 , respectively.

## 4. Discussions

In this paper, we introduced a closed-loop control framework designed to predictively control the peak Achilles tendon force during hopping via a parallel actuator. The proposed controller demonstrated robustness in delivering optimal assistive torques to the target MTU across a spectrum of assistance levels required by the user. Moreover, it exhibited the ability to adapt to the contractile speed characteristics of different muscle fiber phenotypes ranging from slow to fast-twitch, showcasing resilience to variations in muscle type while the controller was aware of the changes. However, this was not the case when the controller was not aware of the changes in the muscle fiber phenotype changes.

### 4.1. Modeling

When damping was incorporated into the conventional Hill-type muscle model, it became feasible to obtain a close-form solution for the tendon force in the form of ODE. As a result, the very low computational time of each frame of the model, 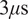 , allowed us to use the model for predicting the future tendon force, knowing the muscle actuation of the future.

In the combined MTU-exoskeleton model, we made the assumption of a single lumped MTU instead of considering individual plantarflexing MTUs (Robertson et al., 2014). Also, the influence of dorsiflexors during hopping was disregarded. Moreover, we assumed a constant moment arm and that the exoskeleton actuation is applied at the same location as the Achilles tendon force. Future work will focus on extending and refining this model to incorporate more detailed representations of the MTU and varying moment arm dynamics.

### 4.2. Control

The predictive framework outlined in this study exhibits certain limitations. Primarily, the internal model of this controller is applicable solely within the range where the normalized F-V relation is linear (refer to Figure 1c). Moreover, we made the assumption of predetermined muscle excitation/activation patterns. To enhance the practical utility of this control framework, a crucial strategy involves incorporating a muscle activation predictor into the algorithm. A significant first step toward this integration could involve leveraging muscle synergies, as discussed in Gonzalez-Vargas et al. (2015); Li et al. (2024). As the activation does not change in response to the assistance provided by the controller, the MTU force is regulated through the manipulation of the MTU contractile mechanics, specifically adjusting the muscle fiber length ( 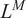 ) and MTU length ( 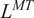 ) using equations (4) and (5), respectively.

#### 4.2.1. Different prediction horizons and levels of robotic support

It was demonstrated that while small initial horizon values ( 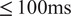 ) in the NMPC algorithm did not effectively constrain the peak Achilles tendon force below the threshold, they did result in a decrease from 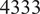 to 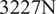 . As the initial horizon increased, the controller began activating earlier, mitigating the risk of surpassing the threshold and allowing for a more gradual increase in actuator activation. However, larger initial horizons, although more likely to maintain the peak tendon force within the limits, can lead to greater deviation from real-world applications in the presence of modeling uncertainties. Furthermore, larger control horizons result in increased computational time required for the controller. In all future simulations, the 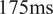 initial horizon was implemented. When possessing the 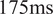 initial horizon, the computational time ( 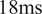 ) stays within the limits of MTU’s physiological electromechanical delay.

Gradually adjusting the support level offered by the exoskeletons can provide benefits. It can aid potential patients in regaining strength and mobility at their preferred pace, enhances the ability of elderly users to carry out daily activities with increased ease and independence, and assists individuals or athletes in optimizing their performance while mitigating the risk of fatigue-related injuries. Consequently, a crucial characteristic of the controller would be its robustness in delivering diverse levels of support without necessitating any modifications to the controller itself. By demonstrating an RMSE of 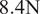 for the peak MTU force and the threshold, the NMPC framework exhibited an acceptable performance in this aspect, highlighting its ability to provide adaptive assistance promptly.

#### 4.2.2. Changes in muscle fiber phenotypes

By accommodating diverse muscle phenotypes, we can extend assistance to a wider range of users with distinct physiological characteristics. The control of different muscle phenotypes enables the exoskeleton to enhance performance across various activities, spanning from rapid bursts of activity to sustained exertion. Additionally, due to the dynamic nature of muscle adaptation, transitional isoforms emerge as muscles possess the capacity to transition between these primary isoforms in long-term (Wisdom et al., 2015). However, in short-term, a more pertinent aspect is the recruitment of different motor units that innervate diverse fiber types, particularly evident during prolonged fatiguing exercises. Consequently, having a controller that robustly adapts to changes in muscle fiber phenotype over time would be advantageous.

When the controller was informed about changes in muscle fiber types, it exhibited robustness against various muscle fiber phenotypes by effectively maintaining the peak tendon force below the threshold, with an RMSE of the maximum tendon force of less than 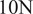 . However, operating without knowledge of the muscle fiber phenotype changes and employing muscle fiber types with slower twitch responses in the controller compared to the primary model, the controller aimed to regulate the maximum tendon force near the threshold. In some instances, it did not entirely succeed but still managed to keep it in proximity to the threshold.

This scenario differed when a muscle fiber with a faster twitch response was utilized in the controller. In such cases, if the 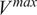 in the controller exceeded that of the model, the controller triggered activation prematurely, resulting in the tendon force falling significantly below the threshold, potentially compromising user comfort in real-world scenarios.

As a limitation of our work, it should be noted that in our simulations, the muscle activation remains unchanged in response to the provided assistance, which differs from real-world scenarios. However, as NMPC functions as a feedback control system, it continuously updates its state and comprehension of activation in real time. Furthermore, different muscle fiber phenotypes exhibit variations not only in 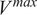 but also in 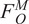 and activation dynamics. However, for the scope of this research, our emphasis was specifically on modulating the 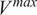 parameter while overlooking changes related to the other mentioned parameters. Furthermore, muscles typically encompass a mix of motor units with diverse fiber types; however, in this study, we simplified by treating the entire muscle as a single muscle fiber type.

In future work, the existing control framework will be deployed for controlling MTU loads for both hopping and walking scenarios (Nabipour et al., 2024), in addition to being employed for controlling trunk exoskeletons during weight-lifting tasks (Li et al., 2024). In this setup, instead of relying on feedback solely from the main model (hopping model in the context of this study), the lengths of the musculotendon units ( 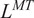 ) and tendon forces will be obtained in real-time from kinematic and EMG data collected from the user. These data will then be processed using the CEINMS real-time toolbox to derive MTU lengths and tendon forces.

## 5. Conclusion

This paper presents a pioneering framework for closed-loop control of peak tendon force within a simulated human ankle joint system equipped with parallel exoskeleton actuation. While previous research in lower limb wearable exoskeleton and exosuit control has primarily focused on reducing metabolic costs or compensating for biological joint torques, our work addresses the critical need for direct control over MTU dynamics. By integrating NMPC with a computationally efficient model encompassing Hill-type MTU contraction dynamics and ankle joint motion with parallel exoskeleton actuation, we bridge a significant gap in wearable robotic control.

Our approach demonstrates robustness across various support levels and diverse MTU fiber phenotypes when the controller is aware of the muscle fiber types, ranging from slow-twitch type I to fast-twitch type IIx. However, the study highlights that this framework is not entirely resilient to changes in muscle fiber phenotypes when the controller lacks awareness of these variations. By effectively managing peak Achilles tendon force under diverse simulated conditions, such as cyclic force production in ankle plantarflexors, our framework sets the stage for future wearable robots capable of delivering precise support tailored to specific MTUs. The computational efficiency of our model, with microsecond-level computation times and resilience to different muscle contraction velocities, underscores its promise for real-world applications.

## Data Availability

The data utilized in this research are sourced from previous studies, as cited in the article. No experimental data were generated specifically for this research project.
